# Spin-based magnetic random-access memory for high-performance computing

**DOI:** 10.1093/nsr/nwad272

**Published:** 2023-10-25

**Authors:** Kaiming Cai, Tianli Jin, Wen Siang Lew

**Affiliations:** School of Physics, Huazhong University of Science and Technology, China; Interuniversity Microelectronics Centre (IMEC), Belgium; School of Physical and Mathematical Sciences, Nanyang Technological University, Singapore; School of Physical and Mathematical Sciences, Nanyang Technological University, Singapore

## Abstract

Spin-based memory technology is now available as embedded magnetic random access memory (eMRAM) for fast, high-density and non-volatile memory products, which can significantly boost computing performance and ignite the development of new computing architectures.

Memory serves as a critical component in today's electronic systems for data storage and processing. In traditional computer architectures, the logic and memory units are physically separated, due to the performance gap in operational speed and capacity among memories, resulting in the fundamental limitation of the von Neumann computers. Moreover, with the evolution of CMOS technology nodes, transistors become smaller and smaller, to improve the operational speed, area density and energy efficiency, while supplying lower driver currents. However, the mainstream technologies, such as embedded-Flash and SRAM, are facing significant scaling and power consumption issues. A denser and more energy-efficient embedded memory would be highly desirable, specifically for advanced technology nodes of 14 nm or beyond. In contrast to conventional electronic devices, manipulating electric charges in non-magnetic semiconductors to process information, spintronic devices are based on the spin of electrons, offering innovative computing solutions. To incorporate spintronics into the existing mature semiconductor technology, the spin-based devices are generally designed with a core structure of a magnetic tunnel junction, which functions as magnetic random access memory (MRAM).

Over the past two decades, various emerging magnetic memories, such as spin-transfer torque (STT), spin-orbit torque (SOT), voltage-control magnetic anisotropy (VCMA), and racetrack MRAMs have been proposed with prospects such as fast speed, non-volatility, excellent endurance, good scalability, and compatibility with CMOS technology [1]. Following conventional computer architectures, MRAMs could be the potential replacements of the current memories in the memory hierarchy, primarily due to their superior speed and density (as shown in Fig. 1). Moreover, features such as non-volatility, stochasticity, and oscillations in MRAM, provide new feasibility for novel computing to solve combinatorial optimization problems, which are notoriously difficult for conventional computers. Differing from conventional digital computing hardware, spintronic devices pave the way for advancements such as spin oscillator-based computing, in-memory computing, probabilistic computing, and domain-wall logic [2–5] for faster and more energy-efficient computing with better scalability.

**Figure 1.**
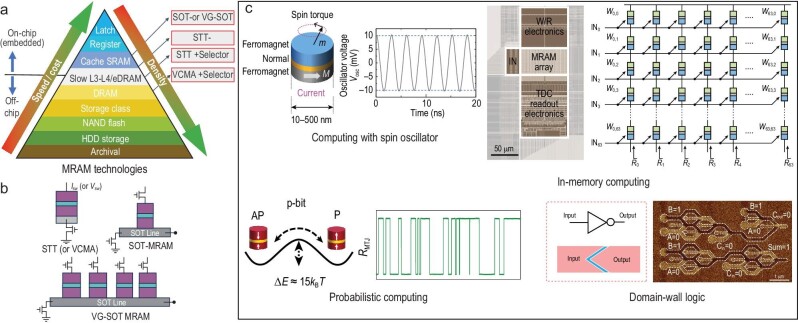
Non-volatile magnetic memories. (a) MRAM technology and proposed memory hierarchy by spintronics solutions. (b) MRAM architectures: two terminal STT (or VCMA)-MRAM with high density and nanosecond write latency for slow cache (L3 or L4) or DRAM applications; three terminal SOT or multi-bit VG-SOT MRAM operate at sub-ns regimes with high endurance for low-level cache replacements [1]. (c) Novel computing by spin-based devices: nano oscillator-based neuromorphic computing [2]. MRAM crossbar array architectures for in-memory computing [3]. Stochastic MTJ for probabilistic computing [4]. Magnetic domain-wall logic [5]. Approaches by nano spintronic oscillators or stochastic MTJs are based on magnetization dynamics. Domain-wall logic promises better scalability and lower energy consumption, taking the benefits of fast motion, high density and non-volatility of magnetic domain walls. Panels adapted with permission from: (c), refs [2–5]. Springer Nature Limited.

Among the MRAMs, STT-MRAM has become a mature technology with the major foundries announcing mass production of embedded STT-MRAM products [6]. Some bottlenecks encountered in previous iterations could be potentially eliminated by using these MRAMs. For instance, one latest news item reported that NXP and TSMC delivered the industry's first automotive-embedded STT-MRAM. Together with the NXP’s automotive processors, this MRAM can update 20 MB of code in around 3 seconds, while it takes ∼1 minute with flash memories. The embedded MRAM significantly minimized downtime and accelerated computing speeds. Moreover, STT-MRAM offers inherently high immunity to magnetic flux and radiation, mitigating the need for radiation shielding during space exploration. More recently, IBM demonstrated a modified STT-MRAM with a double spin-torque for 300 ps switching time, potentially for last-level cache applications [7].

Alternatively, SOT-MRAM has drawn more interest in MRAM technology development due to the improved speed and endurance than STT-MRAM. Owing to its sub-ns switching time and high endurance (>10^12^ cycles) [8], it is, in principle, applicable for all-level caches. However, the three-terminal configuration and high write current in SOT-MRAM adversely impact its reliability and bit-cell area [9]. Adopting a perpendicular magnetization in the free layer of SOT-MRAM improves the scaling potential. However, the necessity of an external magnetic field for write operation limits its implementation in practical applications. Although field-free switching has been realized from a built-in magnet, hybrid switching from an interplay of STT and SOT, or exchange bias field [9–11] in some specific systems, a universal solution for field-free SOT-MRAM is still highly desirable. To further reduce the switching energy, the VG-SOT MRAM scheme, combining SOT and VCMA effects in one system, enables lower switching currents and selective operations between multi-pillar MTJ devices for high-density integration [9]. The multiple MTJs enable multi-level SOT-MRAM devices, making it promising for accurate analog in-memory computing. A pure VCMA-driven MRAM, named VCMA-MRAM, has also been demonstrated with low power and ultrafast switching voltage. All the MRAMs may enhance computing performance with faster speed and lower energy consumption. Moreover, computing based on magnetic devices offers unclonable functions as the root of trust for hardware security [12,13]. The inherent non-volatility of MRAMs can also be leveraged to perform Boolean logic computing in memory [3].

Different from the previous synapse with determined plasticity, the switching probability profile of an MTJ could be tuned by external excitations, such as current pulses and magnetic fields. The stochastic switching behavior of MTJ leads to binary stochastic neurons in a stochastic neural network [4]. An alternative way to realize the stochasticity is by employing a free layer with a low energy barrier. An MTJ cell with intrinsic randomness, known as probabilistic bit (p-bit), removes energy consumption from write operations. The p-bit naturally fluctuates between ‘0’ and ‘1’, which bridges the gap between classical bits (either ‘0’ or ‘1’) and quantum bits (superposition of ‘0’ and ‘1’). Systems of p-bits can address some nondeterministic polynomial-time hard (NP-hard) problems that might otherwise require quantum computing. Recently, there have been demonstrations of using probabilistic computing to solve integer factorization [4,14] or quantum annealing problems [15], which are challenging to classical computers. The MRAM based p-bits, which operate at room temperature and are optimized for large-scale integration, become even more attractive.

Spin-based magnetic memories provide considerable benefits over conventional memories. Good scalability of individual spintronic devices is critical for large-scale integration. The MTJ with perpendicular anisotropy is particularly notable for its thermally stable properties even at minuscule sizes. However, MRAMs present their own set of challenges. Existing MRAMs have a relatively small TMR ratio (typically ∼170%), leading to a narrow read margin or slow read operations. Developing a crossbar array on the chip level remains a challenge due to the low resistance of MRAM [3]. Moreover, further down-scaling of MTJ leads to some penalties in data retention, device variation, write margin and stray field issues [1,16]. Optimizations of film stack and integration process engineering are still in demand.

In summary, MRAMs could significantly enhance computing performance by replacing conventional memories. Computing performance could easily be improved from the superior performance in operational speed and integration density of MRAMs under the traditional architectures. Moreover, due to the non-volatility and inherent stochasticity, magnetic memories can further enable new types of computing, including in-memory computing, neuromorphic computing, and probabilistic computing. Their advancement might change the current computer architecture or extend the capability with new computing schemes.
